# Comprehensive analysis of lncRNA‐associated ceRNA network reveals novel potential prognostic regulatory axes in glioblastoma multiforme

**DOI:** 10.1111/jcmm.18392

**Published:** 2024-06-12

**Authors:** Maryam Bazrgar, Seyed Amir Mirmotalebisohi, Mohsen Ahmadi, Parisa Azimi, Leila Dargahi, Hakimeh Zali, Abolhassan Ahmadiani

**Affiliations:** ^1^ Neuroscience Research Center Shahid Beheshti University of Medical Sciences Tehran Iran; ^2^ Student Research Committee, School of Advanced Technologies in Medicine Shahid Beheshti University of Medical Sciences Tehran Iran; ^3^ Cellular and Molecular Biology Research Center Shahid Beheshti University of Medical Sciences Tehran Iran; ^4^ Department of Medical Genetics, Faculty of Medicine Shahid Beheshti University of Medical Sciences Tehran Iran; ^5^ Neurobiology Research Center Shahid Beheshti University of Medical Sciences Tehran Iran; ^6^ Department of Tissue Engineering and Applied Cell Sciences, School of Advanced Technologies in Medicine Shahid Beheshti University of Medical Sciences Tehran Iran

**Keywords:** AL161785.1, glioblastoma multiforme, LINC02611, miR‐139‐5p, miR‐433‐3p, MS4A6A, PCED1B‐AS1

## Abstract

Deciphering the lncRNA‐associated competitive endogenous RNA (ceRNA) network is essential in decoding glioblastoma multiforme (GBM) pathogenesis by regulating miRNA availability and controlling mRNA stability. This study aimed to explore novel biomarkers for GBM by constructing a lncRNA‐miRNA‐mRNA network. A ceRNA network in GBM was constructed using lncRNA, mRNA and miRNA expression profiles from the TCGA and GEO datasets. Seed nodes were identified by protein–protein interaction (PPI) network analysis of deregulated‐mRNAs (DEmRNAs) in the ceRNA network. A lncRNA‐miRNA‐seed network was constructed by mapping the seed nodes into the preliminary ceRNA network. The impact of the seed nodes on the overall survival (OS) of patients was assessed by the GSCA database. Functional enrichment analysis of the deregulated‐lncRNAs (DElncRNA) in the ceRNA network and genes interacting with OS‐related genes in the PPI network were performed. Finally, the positive correlation between seed nodes and their associated lncRNAs and the expression level of these molecules in GBM tissue compared with normal samples was validated using the GEPIA database. Our analyzes revealed that three novel regulatory axes AL161785.1/miR‐139‐5p/MS4A6A, LINC02611/miR‐139‐5p/MS4A6A and PCED1B‐AS1/miR‐433‐3p/MS4A6A may play essential roles in GBM pathogenesis. MS4A6A is upregulated in GBM and closely associated with shorter survival time of patients. We also identified that MS4A6A expression positively correlates with genes related to tumour‐associated macrophages, which induce macrophage infiltration and immune suppression. The functional enrichment analysis demonstrated that DElncRNAs are mainly involved in neuroactive ligand–receptor interaction, calcium/MAPK signalling pathway, ribosome, GABAergic/Serotonergic/Glutamatergic synapse and immune system process. In addition, genes related to MS4A6A contribute to immune and inflammatory‐related biological processes. Our findings provide novel insights to understand the ceRNA regulation in GBM and identify novel prognostic biomarkers or therapeutic targets.

## 1 INTRODUCTION

Glioblastoma multiforme (GBM) is introduced as the most aggressive type of astrocytoma and one of the most frequent types of primary adult brain tumours. Despite the development and improvement of medical interventions, including surgery, radiotherapy, chemotherapy and biotherapeutic methods, the median survival rate has remained around 15 months, probably due to the lack of deep apprehension about the molecular mechanisms involved in the pathogenesis of the GBM.^1^, ^2^ Therefore, most research efforts have been directed towards investigating the aetiology of GBM, which is believed to have significant benefits for effective GBM therapy.^3^ The classification of GBM has been refined over several years through updates to the World Health Organization's (WHO) classification systems. Recently, the WHO 2021 CNS classification (CNS WHO5) divides adult diffuse gliomas grade IV into IDH1 wild‐type glioblastoma and grade IV IDH1‐mutant astrocytoma.^4^

Several research studies have used different systems biology approaches, including protein–protein interaction (PPI) network analysis and gene regulatory networks (GRNs), to decipher the molecular pathology behind various illnesses and conditions.^5^, ^6^, ^7^, ^8^ Some research has stated that GRN disruption is related to developing various diseases, including GBM.^9^, ^10^ Besides, studies have demonstrated a role for both protein‐coding and non‐coding RNAs (ncRNA) in aberrant gene expression and GBM progression^11^, ^12^, ^13^; However, their regulatory relationships and functions are still largely unknown. The ncRNA dysregulation is reported to play essential roles in disease pathogenesis through dysregulating messenger RNA (mRNA) stability and translation. In 2011, a theory for how ncRNAs function was proposed under the title of competitive endogenous RNA (ceRNA), indicating circular RNAs, lncRNAs and pseudogenes act as miRNA sponges and control the availability of endogenous miRNAs to bind to their target mRNAs.^14^ This way, lncRNAs can form a ceRNA regulatory network to modulate mRNA expression and regulate the tissue protein levels. Many studies have shown that the ceRNA regulatory axes have a powerful effect on the progression and pathogenesis of many cancers, including GBM. For instance, Wu et al. have indicated that the miR155HG/miR‐185/ANXA2 axis is likely to contribute to the progression of glioblastoma.^15^ Another study unearthed that the OXCT1‐AS1/miR‐195/CDC25A axis has a role in glioblastoma tumorigenesis.^16^ The construction and analysis of ceRNA networks is an attractive approach to identifying critical genes, may provide several clues to uncover the pathogenesis and probably has the potential to discover novel therapeutic targets for GBM. In this study, to further understand the potential role of protein‐coding and non‐coding RNAs in GBM, ceRNA regulatory network principles and bioinformatics approaches were applied to construct and analyse the LncRNA‐miRNA‐mRNA regulatory network. This study may illuminate the underlying mechanisms of GBM pathogenesis and provide novel prognostic and therapeutic biomarkers for GBM.

## 2 METHODS

### 2.1 Study design

**FIGURE 1:**
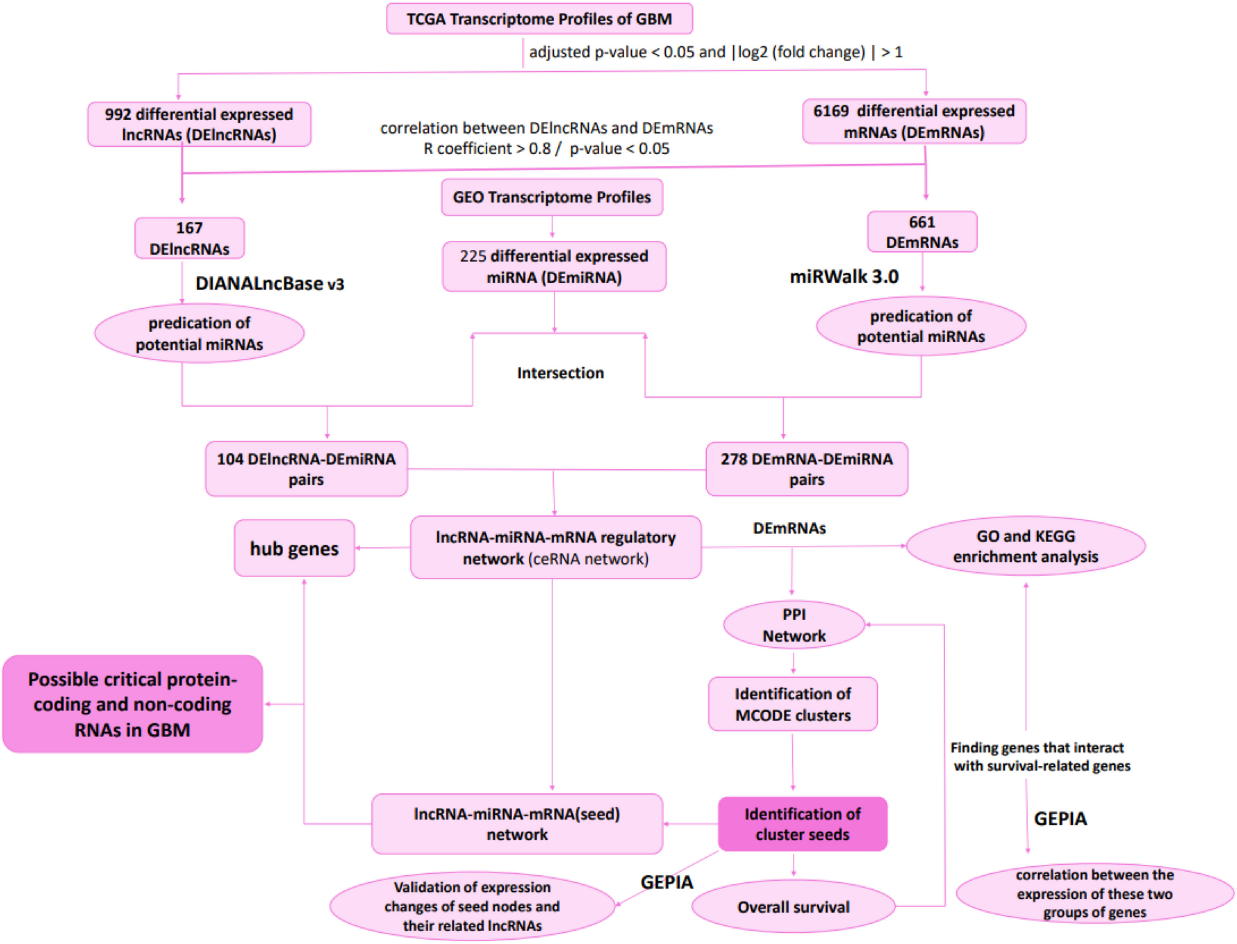
Workflow of the construction of lncRNA‐associated ceRNA network. ceRNA, competitive endogenous RNA; DE, differentially expressed; GEO, Gene Expression Omnibus; GEPIA, Gene Expression Profiling Interactive Analysis; GO, Gene Ontology; KEGG, Kyoto Encyclopedia of Genes and Genomes; lncRNA, long non‐coding RNAs; miRNA, microRNA; PPI, protein–protein interaction network; TCGA, The Cancer Genome Atlas.

All databases have been used to analyse data, GBM was labeled as IDH1mut/wt glioma grade IV (2016 WHO brain tumour classification). We analysed RNA expression profiles in IDH1mut/wt glioma grade IV (GBM) patients^4^ from The Cancer Genome Atlas (TCGA) database to screen the differentially expressed lncRNAs (DElncRNAs) and mRNAs (DEmRNAs) that are associated with GBM. The GSE65626 data set was used for microRNA expression profile analysis to identify differentially expressed miRNAs (DEmiRNA). Then, the potential target miRNAs of the DElncRNAs and putative interactions between the acquired miRNAs and DEmRNAs were identified through DIANALncBase v3 and miRWalk 3.0, respectively. Predicted miRNAs were intersected with DEmiRNAs, and if both DEmRNA and DElncRNA that were positively correlated (*R* coefficient > 0.8) were targeted by the same miRNA with opposite expression, this lncRNA‐miRNA‐mRNA interaction was selected for the construction of ceRNA regulatory network. In the following, we also constructed a PPI network of DEmRNAs in the ceRNA network and lncRNA‐miRNA‐mRNA (seed) network by combining bioinformatics and correlation analyses to find the critical protein‐coding and non‐coding RNAs in GBM. An outline of the workflow of this study is summarized in Figure 1.

### 2.2 TCGA data collection, pre‐processing and analysis of differentially expressed mRNAs and LncRNAs

We employed the TCGAbiolinks R package^17^ to download RNAseq data of 169 GBM (IDH1mut/wt glioma grade IV) and five normal brain samples from TCGA database (http://portal.gdc.cancer.gov). The data set included quantitative expression levels of mRNAs and lncRNAs in GBM and control samples. Clinical data of all samples are summarized in File S1. All TCGA data were processed utilizing the statistical programming language R. For pre‐processing, count data were first filtered by the DGElist and filterByExpr functions in the edgeR Bioconductor package (https://www.bioconductor.org/packages/devel/bioc/html/edgeR.html), then the calcNormFactors and voom functions from limma Bioconductor package (https://www.bioconductor.org/packages/devel/bioc/html/limma.html) were applied to normalize the data. We also used the limma Bioconductor package in R language (https://www.bioconductor.org/packages/devel/bioc/html/limma.html) to demonstrate differentially expressed genes (DEGs) between GBM samples and normal counterparts, including mRNAs (DEmRNAs) and lncRNAs (DElncRNAs), respectively. The mRNAs and lncRNAs were separated via the HUGO Gene Nomenclature Committee (HGNC) database (https://www.genenames.org/) in the R software. The HGNC is a comprehensive resource for approved human gene nomenclature containing ~42,000 gene symbols, names and 1300+ gene families and sets. The DElncRNAs and DEmRNAs were filtered according to the cut‐off criteria of adjusted *p*‐value < 0.05 and |log2 (fold change)| > 1. Subsequently, the EnhancedVolcano package in the R software was utilized to visualize all the DEGs with a volcano plot.

### 2.3 GEO data collection and analysis of differentially expressed miRNAs

We retrieved the human microRNA expression profiles (accession number: GSE65626) of GBM from the National Center of Biotechnology Information (NCBI) Gene Expression Omnibus (GEO) (https://www.ncbi.nlm.nih.gov/geo/). The samples were derived from the three tumour tissues and three normal tissues. The microarray platform used to analyse these data was GPL19117 [miRNA‐4] Afymetrix Multispecies miRNA‐4 Array. We used the GEO2R (http://www.ncbi.nlm.nih.gov/geo/geo2r/) to normalize expression data and filter differentially expressed miRNAs (DEmiRNA) between normal and tumour samples. The DEmiRNAs were filtered according to the cut‐off criteria of *p*‐value < 0.05 and |log2 (fold change)| > 1. GEO2R was utilized to visualize normalized expression data and DEmiRNAs with boxplot and volcano plot, respectively.

### 2.4 Prediction of target miRNAs of DElncRNAs and miRNA‐DEmRNAs interactions

The potential target miRNAs of the DElncRNAs were identified through DIANALncBase v3 (https://diana.e‐ce.uth.gr/lncbasev3/interactions). This platform serves as a reference repository, offering experimentally supported miRNA targets on long non‐coding RNA (lncRNA). To ensure a robust selection, we considered both low and high miRNA confidence levels for the lncRNA‐miRNA interactions within the database. Notably, DIANA‐LncBase v3 catalogues approximately ~500,000 entries, corresponding to ~240,000 unique tissue and cell‐type‐specific miRNA‐lncRNA interactions. The incorporated interactions are defined by 15 distinct low‐/high‐throughput methodologies, corresponding to 243 different cell types/tissues and 162 experimental conditions.^18^ The putative interactions between the acquired miRNAs and DEmRNAs were determined using miRWalk 3.0^19^ (http://mirwalk.umm.uni‐heidelberg.de/), which integrated the prediction results of both TargetScan^20^ and miRDB,^21^ and the score ≥ 0.95 was considered as the threshold for the prediction analysis in miRWalk database.

### 2.5 Construction of the lncRNA‐associated ceRNA network and topological analysis

LncRNA‐miRNA‐mRNA interactions were identified as a potential ceRNA triple based on the following criteria: (1) The Pearson's correlation coefficients (PCCs) between each DElncRNAs–DEmRNAs pair in GBM were calculated. The DElncRNAs–DEmRNAs pairs whose *R* coefficient of the PCC was more significant than 0.8 with a *p*‐value <0.05 were considered for the lncRNA‐associated ceRNA network. (2) Predicted miRNAs were intersected with DEmiRNAs, and only the interaction of DEmiRNA and DEmRNA with opposite expressions was included in the present study. (3) If both DEmRNA and DElncRNA that were positively correlated (*R* coefficient > 0.8) were targeted by the same miRNA with opposite expression, this lncRNA‐miRNA‐mRNA interaction was selected for the construction of the ceRNA regulatory network.

To give an insight into the roles of lncRNAs in the ceRNA network, we assembled all the potential co‐dysregulated competing triples to build the upregulated lncRNAs‐downregulated miRNAs‐overexpressed mRNAs network and the lower expressed lncRNAs‐upregulated miRNAs‐downregulated mRNAs network. Finally, regulatory networks constructed from these interactions were visualized using Cytoscape V 3.9.0.

Topological analysis is essential to discover information in complex data sets. To study the geometric relationships between the data nodes, both up and down regulatory networks were merged, and the node degree of each node, which is a critical network topological feature, was computed. Then, the top 10% of the nodes with a high degree score were considered the hub nodes in the regulation network, which were more likely to play a crucial role in GBM.

### 2.6 PPI Network construction, identification of MCODE clusters and seed nodes

To further explore the association between DEmRNAs, PPI network analysis of DEmRNAs in the lncRNAs‐miRNAs‐mRNAs network was performed using the online database STRING with an interaction score of 0.4 as the threshold. Next, we utilized cytoscape V 3.9.0 to construct, visualize and analyse the PPI network. The Molecular Complex Detection (MCODE) plug‐in in Cytoscape was used to identify the MCODE clusters (Degree cut‐off ≥5, node score cut‐off ≥2, K‐core ≥2, and max depth = 100). The seed nodes in the cluster were considered core genes. In a PPI network, seed nodes refer to a set of proteins known or hypothesized to be involved in a specific biological process or pathway of interest. Seed nodes are often used in network‐based approaches to prioritize candidate genes or proteins for further experimental validation or to study the global behaviour of the network.^22^, ^23^, ^24^

### 2.7 Construction of the lncRNA‐miRNA‐mRNA(seed) network

To identify the relationship among DElncRNAs, DEmiRNAs and seed nodes, the seeds mentioned above were mapped into the primary lncRNA‐miRNA‐mRNA network, and the relevant DElncRNAs and DEmiRNAs were also extracted to construct the network using the Cytoscape software.

### 2.8 Functional enrichment analysis

We elucidated the underlying mechanisms of the obtained DElncRNAs in GBM by performing the Gene Ontology (GO) and Kyoto Encyclopedia of Genes and Genomes (KEGG) pathway analysis by uploading the name of DEmRNAs in the ceRNA network to the Database for Annotation, Visualization and Integrated Discovery (https://david.ncifcrf.gov/summary.jsp). To further investigate the potential functions of survival‐related genes of GBM, GO and KEGG pathway enrichment analyses were also performed on the genes that interacted with survival‐related genes in the PPI network. The GO items consist of three parts: biological process (BP), molecular functions (MF) and cellular components (CC). Adjusted *p*‐value < 0.05 was considered statistically significant.

### 2.9 Validation of expression changes in seed nodes and their related lncRNAs

The Gene Expression Profiling Interactive Analysis (GEPIA) tool (http://gepia.cancer‐pku.cn/) was used to combine the brain gene expression profile from the GTEx database^25^ and TCGA paracancer tissue data to validate the aberrant expression of seed genes and their related lncRNAs. Furthermore, we validate seed gene expressions at the protein level using the CPTAC (Clinical Proteomic Tumour Analysis Consortium) data set of the UALCAN portal (http://ualcan.path.uab.edu/analysis‐prot.html).^26^

### 2.10 Overall survival analysis

To determine the prognosis of GBM patients with differentially expressed mRNA signatures, overall survival (OS) curves of seed nodes in MCODE clusters were analysed using the GSCA database (http://bioinfo.life.hust.edu.cn/GSCA/#/) with a threshold of log‐rank *p* < 0.05. GSCA utilizes mRNA expression and clinical outcome data of 33 cancer types from the TCGA database and the University of California, Santa Cruz (UCSC).^27^ GSCA employs the median mRNA value to categorize tumour samples into high‐ and low‐expression groups. The R package survival was used to model these two groups' survival time and status. Cox Proportional‐Hazards model and Logrank tests were conducted for each gene in every cancer.

### 2.11 Evaluation of the correlation between immune infiltration and survival‐related gene expression

The correlation between gene mRNA expression and immune cells' infiltrates in GBM was analysed using the GSCA database.^27^ This platform estimates infiltrates of 24 immune cells in the 33 cancer types' immune microenvironment through ImmuCellAI. ImmuCellAI is a gene set signature‐based method for precisely evaluating the abundance of 24 immune cell types from RSEM‐normalized mRNA expression data downloaded from the TCGA database.^28^ The 24 immune cells comprise dendritic cells (DCs), B cells, monocytes, macrophages, natural killer cells (NK), neutrophils, CD4 T cells, CD8 T cells, natural killer T cells (NKT), gamma delta T cells, CD4 naïve T cells, CD8 naïve T cells, cytotoxic T cells, exhausted T cells, type 1 regulatory T cells (Tr1), natural regulatory T cells, induced regulatory T cells, T helper type 1 (Th1), T helper type 2 (Th2), T helper type 17 (Th17), T follicular helper cell (Tfh), central memory T cells, effector memory T cells and mucosal‐associated invariant T cells (MAIT). Adjusted *p*‐value < 0.05 was considered statistically significant.

## 3 RESULTS

### 3.1 Data pre‐processing and identification of DElncRNAs, DEmiRNAs and DEmRNAs

**FIGURE 2:**
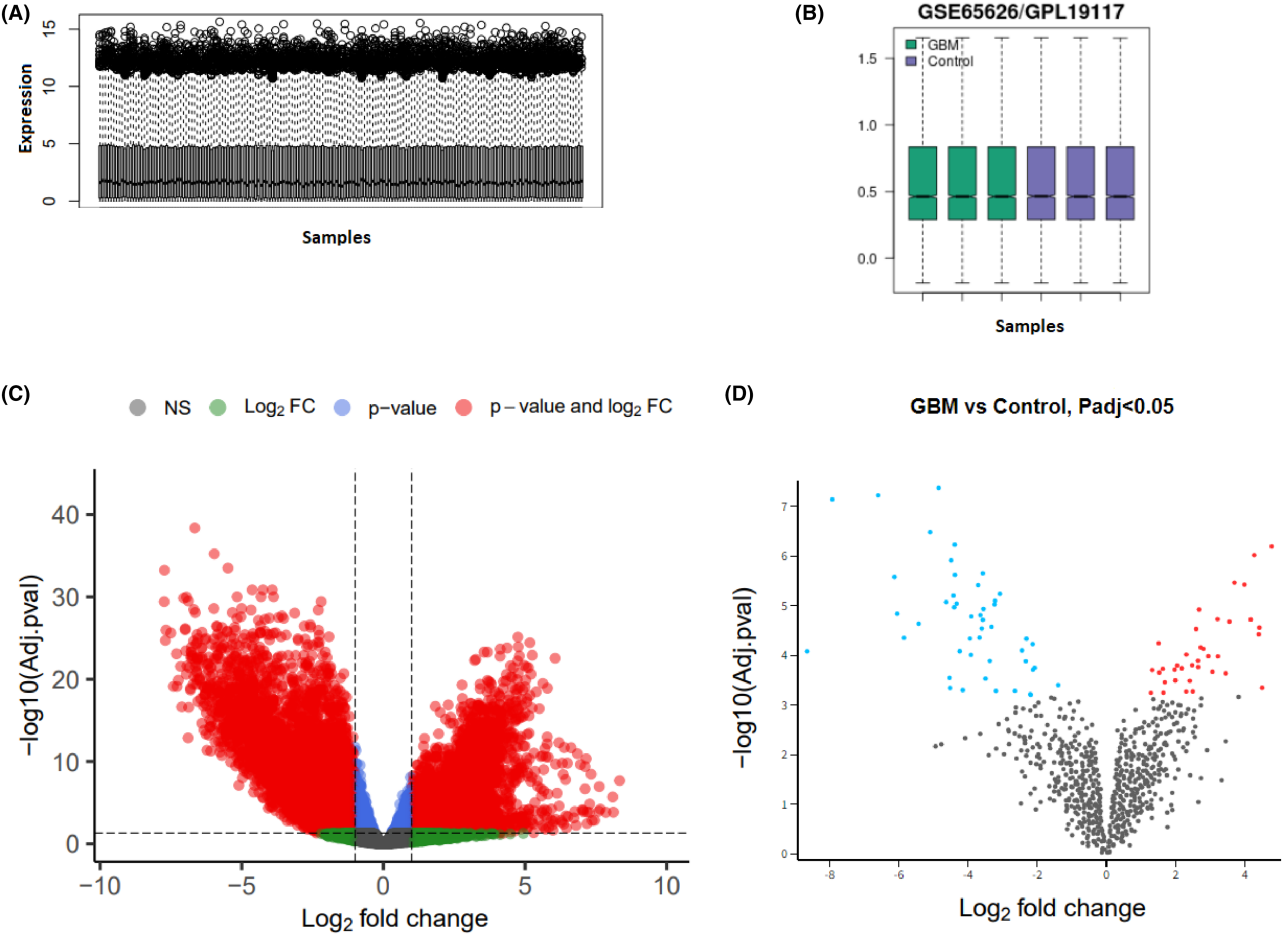
Data quality assessment and identification of differentially expressed genes (DEG). (A) Boxplot of intensity distributions in the normalized RNAseq data. The relative lncRNA and mRNA expression values are comparable among all 174 samples after normalization. (B) Boxplot for the normalized microarray data of microRNA expression profiles. Each box represents a sample. (C) Volcano plot of DElncRNAs and DEmRNAs, (D) Volcano plot of DEmiRNA in Glioblastoma Multiforme.

Figure 2A,B show the normalized RNAseq and microarray data boxplots, respectively. These boxplots show that the expression data quality was reliable and the normalization was sound. Using the normalized RNAseq data shown in Figure 2A, we demonstrated that 6169 mRNAs were differentially expressed, in which 2463 of them were upregulated, and 3706 were downregulated in GBM tissues compared with normal samples (File S2). We also identified 992 differentially expressed lncRNAs; of them, 344 were significantly overexpressed, and 648 were significantly lower expressed in GBM samples compared to the normal tissues (File S3). Based on the microarray data analysis between GBM and normal samples, we obtained 225 DEmiRNAs, including 115 downregulated DEmiRNAs and 110 upregulated DEmiRNAs (File S4). The expression of all DEGs, including DElncRNAs, DEmiRNAs and DEmRNAs, are shown in two volcano plots (Figure 2C,D).

### 3.2 Construction of lncRNA‐associated ceRNA network

**FIGURE 3:**
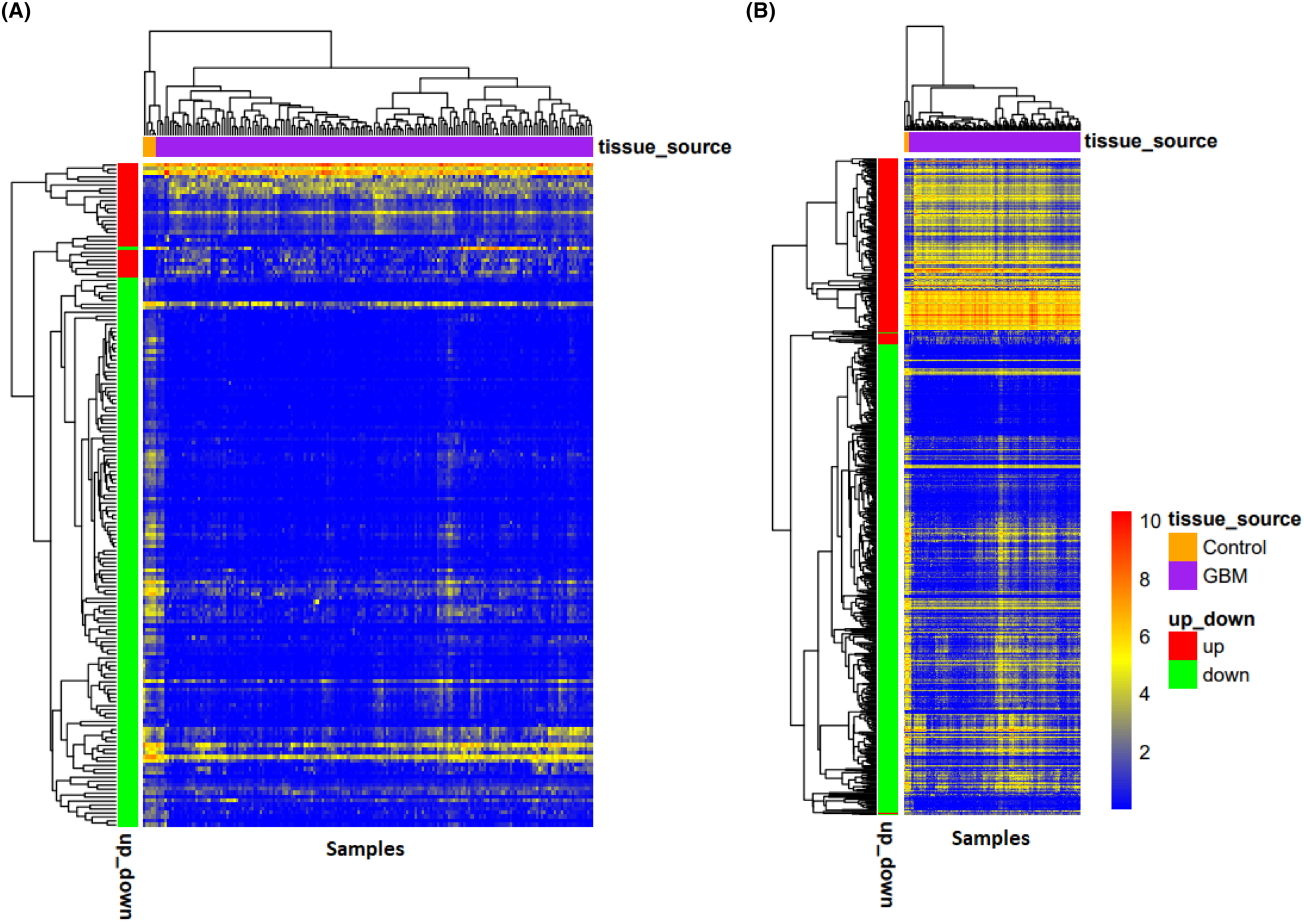
Heatmap of differentially expressed lncRNAs (A) and mRNAs (B) in Glioblastoma Multiforme that were correlated. The horizontal axis shows the names of 174 samples. The vertical axis presents the gene names.

**FIGURE 4:**
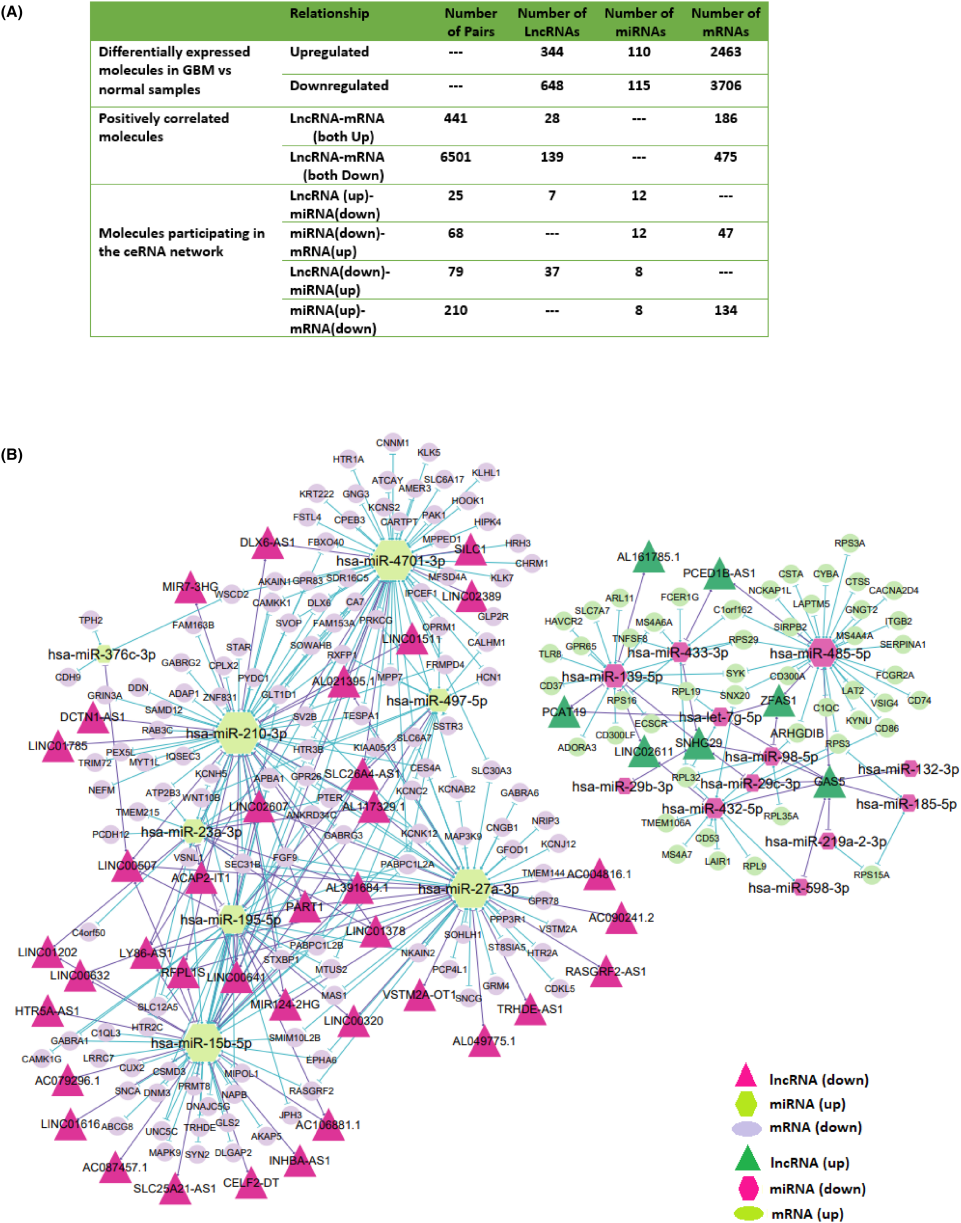
The lncRNA‐associated ceRNA network. (A) The number of relationships and pairs among lncRNAs, miRNAs, and mRNAs in the ceRNA network and the number of differentially expressed molecules and correlated molecules. (B)The ceRNA network was constructed based on identified lncRNA–miRNA and miRNA–mRNA interactions. The networks include down/upregulated lncRNAs, up/downregulated miRNAs, and lower/overexpressed mRNAs in Glioblastoma Multiforme. All node sizes were represented by degree.

When we evaluated the correlation between the acquired DElncRNAs and DEmRNAs, we observed that 167 DElncRNAs had a significant positive correlation with 661 DEmRNAs (File S5). Two heatmaps were constructed to visualize cluster analysis results of the expression of these lncRNAs and mRNAs that were correlated (Figure 3). The identification of target DEmiRNAs for these DElncRNAs and DEmRNAs revealed 104 lncRNA‐miRNA, 278 miRNA‐mRNA and 531 correlated lncRNA‐mRNA pairs. Among these, 25 pairs were associated with upregulated lncRNAs‐downregulated miRNAs, 68 were related to decreased miRNAs‐overexpressed mRNAs, and 67 were related to upregulated/correlated lncRNAs‐mRNAs (File S6). Besides, 79, 210, and 464 pairs were associated with downregulated lncRNAs‐upregulated miRNAs, increased miRNAs‐lower expressed mRNAs, and correlated DElncRNAs‐DEmRNAs with reduced expression patterns, respectively (File S7). The final lncRNA‐associated ceRNA network consisted of 37 DElncRNAs, 12 DEmiRNAs, and 134 DEmRNAs with lowered expression patterns and 7 DElncRNAs, 8 DEmiRNAs, and 47 DEmRNAs with elevated expression profile (Figure 4).

### 3.3 Demonstration of the hub genes in lncRNA‐associated ceRNA network

Utilizing the topological analysis, we determined the top 10% hub genes in established ceRNA network, including two upregulated DElncRNAs (GAS5 and SNHG29), three downregulated DElncRNA (PART1, LINC00507, and LINC00641), seven overexpressed miRNAs (hsa‐miR‐210‐3p, hsa‐miR‐4701‐3p, hsa‐miR‐27a‐3p, hsa‐miR‐15b‐5p, hsa‐miR‐195‐5p, hsa‐miR‐23a‐3p, and hsa‐miR‐497‐5p), six lower expressed miRNAs (hsa‐miR‐139‐5p, hsa‐miR‐485‐5p, hsa‐miR‐432‐5p, hsa‐miR‐433‐3p, hsa‐let‐7 g‐5p, hsa‐miR‐98‐5p), six decreased DEmRNAs (APBA1, PABPC1L2A, HTR3B, SEC31B, GPR26, and PTER) and one increased DEmRNAs (RPL32) (Table 3, File S8).

### 3.4 PPI network construction, identification of MCODE clusters and seed nodes

**FIGURE 5:**
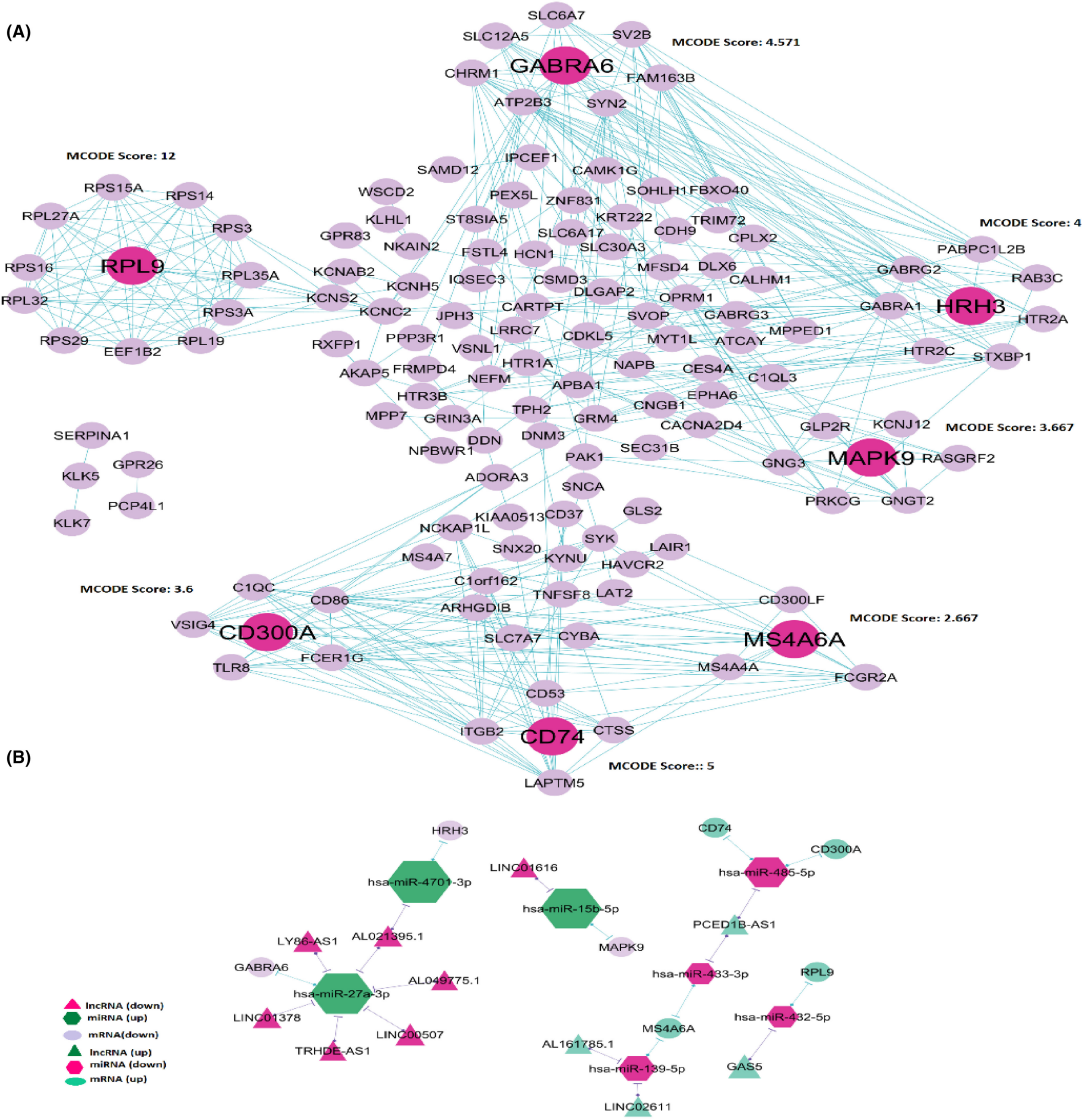
(A) protein–protein interaction network of DE‐mRNAs in lncRNAs‐miRNAs‐mRNAs network. Seven clusters were identified by the MCODE algorithm represented in the PPI network. The seed nodes in each cluster are shown in pink. (B) lncRNA‐miRNA‐seed network. This network consisted of 11 lncRNAs, seven miRNAs, and seven mRNAs.

PPI networks of 181 DEmRNAs in lncRNA‐associated ceRNA network showed 132 nodes and 858 edges (Figure 5A). By utilizing the algorithm of MCODE, seven clusters were identified (Figure 5A). The seven seed genes in the clusters included MS4A6A, CD74, CD300A, RPL9, GABRA6, HRH3 and MAPK9, whose degrees were 12,10, 8, 13, 16, 10 and 2, respectively. MS4A6A, CD74, CD300A and RPL9 expressions were upregulated in GBM compared with the control, and GABRA6, HRH3, and MAPK9 expressions decreased in GBM. These seven genes were considered as functional genes (Table 1).

**TABLE 1:** Seed nodes in the PPI network.

Seed nodes	Degree	logFC	Adj.*p*.val
MS4A6A	12	4.081934	7.31E‐06
CD74	10	2.209184	6.79E‐05
CD300A	8	2.054989	0.001766
RPL9	13	1.448698	0.001613
GABRA6	16	−4.18673	4.86E‐17
HRH3	10	−6.1463	2.56E‐19
MAPK9	2	−2.19850	3.76E‐30

### 3.5 Construction of the lncRNA‐miRNA‐mRNA (seed) network

By mapping the seven seed genes into the preliminary lncRNA‐miRNA‐mRNA network and extracting relevant lncRNAs and miRNAs, a network considered lncRNA‐miRNA‐seed was constructed. This network consisted of 11 lncRNAs (AL161785.1, LINC02611, PCED1B‐AS1, GAS5, LINC01616, AL021395.1, LINC00507, LINC01378, AL049775.1, LY86‐AS1 and TRHDE‐AS1), seven miRNA (hsa‐miR‐139‐5p, hsa‐miR‐432‐5p, hsa‐miR‐433‐3p, hsa‐miR‐485‐5p, hsa‐miR‐27a‐3p, hsa‐miR‐4701‐3p and hsa‐miR‐15b‐5p) and seven mRNA (MS4A6A, CD300A, CD74, RPL9, GABRA6, HRH3 and MAPK9) with 21 edges (Figure 5B). There were 14 lncRNA‐miRNA‐mRNA regulatory axes identified in the network (Figure 5B, File S8).

### 3.6 Evaluation of overall survival for seed nodes

**FIGURE 6:**
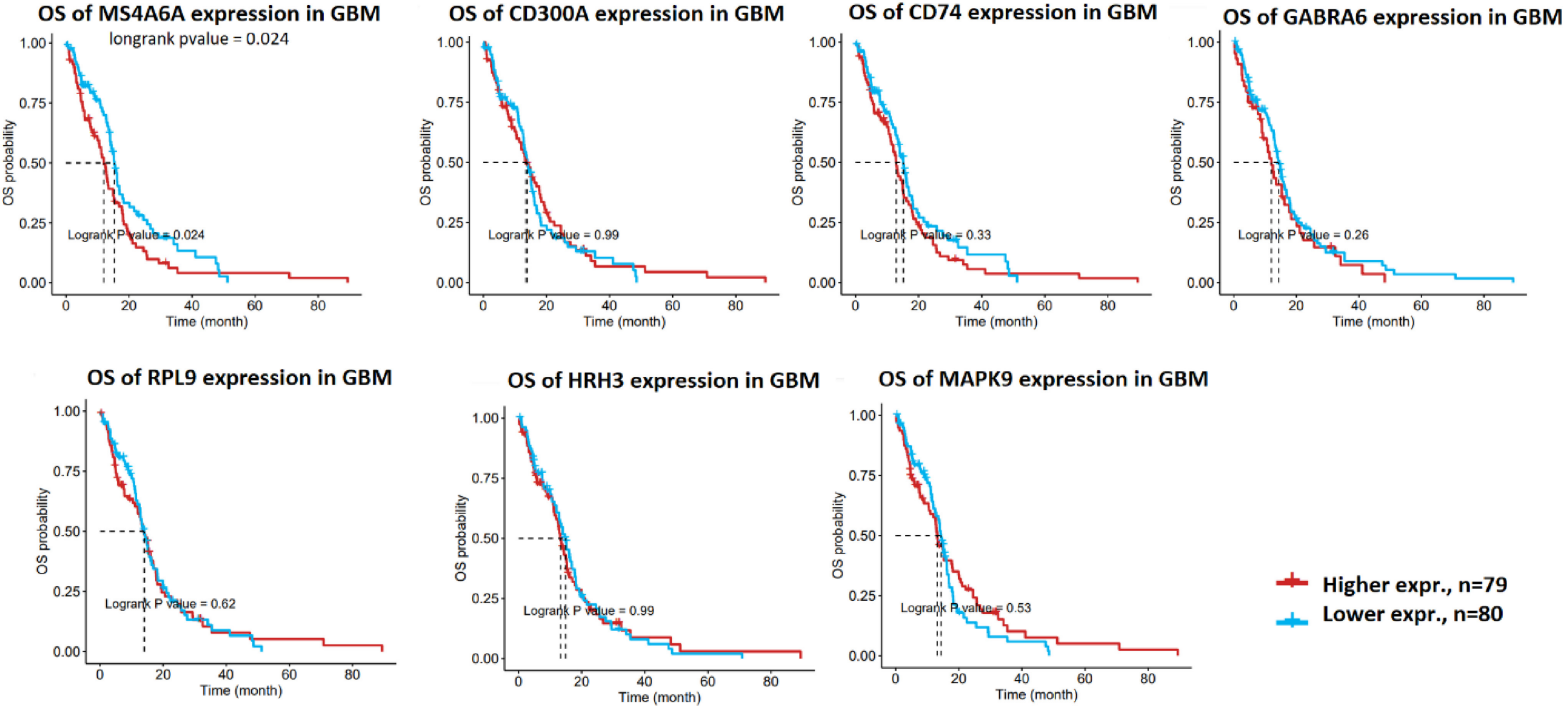
Overall survival (OS) of the seven seed genes (MS4A6A, CD300A CD74, GABRA6, RPL9, HRH3, and MAPK9) from GSCA. GSCA, Gene Set Cancer Analysis.

GSCA was used to assess the seven seed genes' overall survival (OS). Notably, higher expression of Membrane Spanning 4‐Domains A6A (MS4A6A) revealed a significantly poorer OS (hazard ratio = 1.48, log‐rank *p* = 0.024). However, no significant effect was indicated regarding OS for the remaining six seed genes (Figure 6).

### 3.7 Identification of genes that interacted with MS4A6A in the PPI network

**FIGURE 7:**
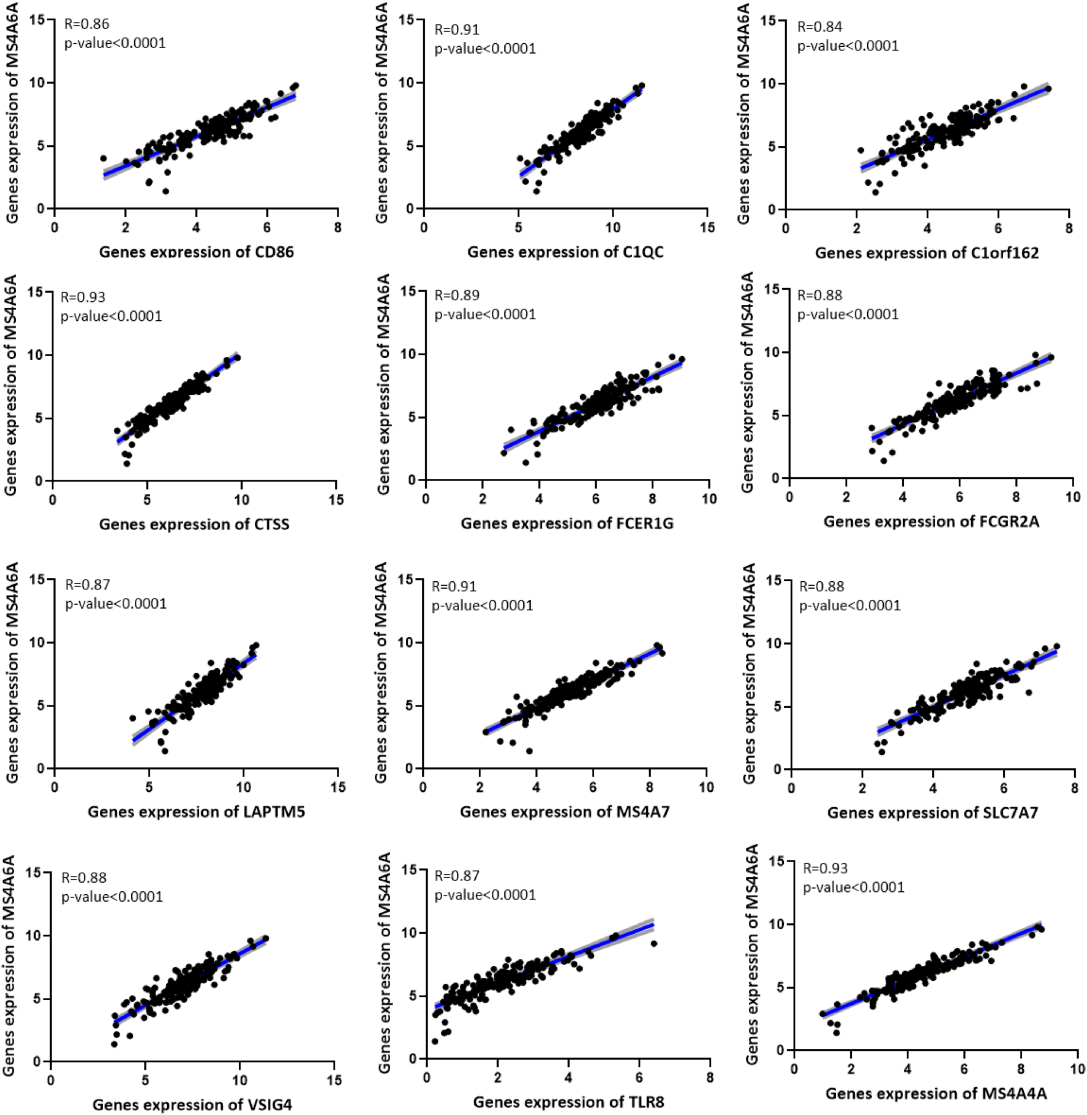
Positive correlation between 12 genes expression (CD86, C1QC, C1orf162, CTSS, FCER1G, FCGR2A, LAPTM5, MS4A7, SLC7A7, VSIG4, TLR8, and MS4A4A) and MS4A6A expression. The connecting line is shown in blue, and error bars are shown in grey.

PPI network demonstrated that C1QC, C1orf162, CD86, CTSS, FCER1G, FCGR2A, LAPTM5, MS4A4A, MS4A7, SLC7A7, TLR8 and VSIG4 might interact with MS4A6A (Figure 5A). Our study showed positive correlation between these 12 genes expression and MS4A6A expression (*R* > 0.8) (Figure 7). These results were validated by the GEPIA database (File S9).

### 3.8 Functional enrichment analysis of DEmRNAs in the ceRNA network and Genes related to MS4A6A

We performed GO and KEGG enrichment analysis of the 181 DEmRNAs in the lncRNA‐associated ceRNA network and selected 10 results from them (Table 2). The significantly enriched BP terms were mainly associated with chemical synaptic transmission, cytoplasmic translation, positive regulation of NIK/NF‐kappaB signalling, negative regulation of MyD88‐dependent Toll‐like receptor signalling pathway, and immune system process. The significantly enriched MF terms were mainly related to neurotransmitter receptor activity, structural constituent of ribosome, protein serine/threonine/tyrosine kinase activity, extracellular ligand‐gated ion channel activity, GABA‐gated chloride ion channel activity, and other functions. Regarding CC, enriched items were mainly synapses, plasma membranes, glutamatergic synapses, ribosomes, neuron projection, etc. KEGG pathway analysis revealed that the DEmRNAs in the ceRNA network were primarily involved in the neuroactive ligand‐receptor interaction, GABAergic synapse, calcium signalling pathway, MAPK signalling pathway, serotonergic synapse, ribosome and other pathways. The results of GO and KEGG analyses can be found in Table 2 and File S10. To investigate the potential functions of MS4A6A in GBM, GO and KEGG pathway enrichment analyses were also performed on the genes that interacted with MS4A6A in the PPI network. The GO analysis of these 12 genes indicated involvement in immune‐related biological processes, including antigen processing and presentation of exogenous peptide antigen via MHC class II, negative regulation of T‐cell proliferation, immunoglobulin‐mediated immune response, inflammatory response such as Toll‐like receptor signalling pathway, positive regulation of interleukin‐4 production, negative regulation of interleukin‐2 production, and positive regulation of NIK/NF‐kappaB signalling. KEGG analysis revealed that genes are associated with systemic lupus erythematosus and Tuberculosis (Table 3, File S10).

**TABLE 2:** GO and KEGG enrichment analysis of the 181 DEmRNAs in lncRNA‐associated ceRNA network.

Category	Term	Count	Genes	*p* Value
BP	GO:0007268~chemical synaptic transmission	21	PRKCG, GABRA1, CHRM1, SLC12A5, GABRA6, HTR1A, HTR2C, CARTPT…	5.01E‐14
BP	GO:0002181~cytoplasmic translation	11	RPS14, RPS15A, RPS16, RPL32, RPS29, RPL27A, RPS3, RPL35A, RPS3A, RPL9, RPL19	4.50E‐09
BP	GO:0007187~G‐protein coupled receptor signalling pathway, coupled to cyclic nucleotide second messenger	7	CHRM1, HRH3, HTR1A, HTR2C, HTR2A, OPRM1, SSTR3	1.81E‐05
BP	GO:0007214~gamma‐aminobutyric acid signalling pathway	5	GABRA1, GABRA6, HTR1A, GABRG3, GABRG2	8.58E‐05
BP	GO:0031623~receptor internalization	5	DNM3, FCER1G, SYK, ITGB2, SNCA	9.01E‐04
BP	GO:0007269~neurotransmitter secretion	4	GRM4, HRH3, STXBP1, SYN2	0.002032
BP	GO:0002376~immune system process	4	FCGR2A, SYK, CD300A, CD300LF	0.013619
BP	GO:0034125~negative regulation of MyD88‐dependent toll‐like receptor signalling pathway	2	CD300A, CD300LF	0.016824
BP	GO:0043303~mast cell degranulation	3	LAT2, SYK, CPLX2	0.01733
BP	GO:1901224~positive regulation of NIK/NF‐kappaB signalling	4	CD86, RPS3, LAPTM5, HAVCR2	0.022257
MF	GO:0030594~neurotransmitter receptor activity	10	GABRA1, CHRM1, HRH3, GABRA6, HTR1A, HTR2C, HTR3B, HTR2A, GABRG3, GABRG2	8.03E‐08
MF	GO:0003735~structural constituent of ribosome	11	RPS14, RPS15A, RPS16, RPL32, RPS29, RPL27A, RPS3, RPL35A, RPS3A, RPL9, RPL19	1.23E‐05
MF	GO:0005230~extracellular ligand‐gated ion channel activity	5	GABRA1, GABRA6, HTR3B, GABRG3, GABRG2	1.90E‐04
MF	GO:0022851~GABA‐gated chloride ion channel activity	4	GABRA1, GABRA6, GABRG3, GABRG2	2.14E‐04
MF	GO:0004993~G‐protein coupled serotonin receptor activity	5	CHRM1, HRH3, HTR1A, HTR2C, HTR2A	2.74E‐04
MF	GO:0005249~voltage‐gated potassium channel activity	5	KCNH5, KCNC2, KCNS2, KCNAB2, HCN1	0.003025
MF	GO:0051378~serotonin binding	3	HTR1A, HTR2C, HTR2A	0.006327
MF	GO:0005516~calmodulin binding	7	PPP3R1, KCNH5, RASGRF2, ATP2B3, AKAP5, CAMK1G, CAMKK1	0.013354
MF	GO:0031681~G‐protein beta‐subunit binding	3	GNG3, GNGT2, OPRM1	0.019307
MF	GO:0004712~protein serine/threonine/tyrosine kinase activity	10	PRKCG, HIPK4, MAPK9, PAK1, EPHA6, CDKL5, SYK, MAP3K9, CAMK1G, CAMKK1	0.022773
CC	GO:0045202~synapse	33	CHRM1, RPL32, KCNC2, HTR2C, CPLX2, RPS15A, RPS16, ATCAY…	5.01E‐18
CC	GO:0005886~plasma membrane	95	CD86, RAB3C, GPR26, CHRM1, KCNC2, GPR65, RASGRF2, ITGB2, MS4A7, HTR2C, SIRPB2, HTR2A, RXFP1, CDH9, MS4A4A…	2.17E‐14
CC	GO:0005887~integral component of plasma membrane	41	CHRM1, KCNC2, GPR65, HTR2C, GPR83, HTR2A, PCDH12, TRHDE, RXFP1, CALHM1, GRM4, HRH3…	3.38E‐11
CC	GO:0045211~postsynaptic membrane	15	GABRA1, CHRM1, GABRA6, KCNC2, IQSEC3, HTR3B, HTR2A, OPRM1, GABRG3, CDH9, GABRG2, DDN, GRIN3A, LRRC7, HCN1	2.25E‐09
CC	GO:0032590~dendrite membrane	8	GABRA1, SLC12A5, GABRA6, KCNC2, AKAP5, OPRM1, GABRG3, GABRG2	3.35E‐09
CC	GO:0098978~glutamatergic synapse	19	NAPB, CHRM1, CDKL5, SLC30A3, SLC6A17, FRMPD4, STXBP1, ATP2B3, HTR2A, CPLX2, SYN2, C1QL3, GABRG2, DNM3, PPP3R1, GRIN3A, APBA1, DLGAP2, HCN1	2.08E‐08
CC	GO:0043005~neuron projection	17	GABRA1, TPH2, EPHA6, SLC12A5, SLC30A3, GABRA6, HTR3B, OPRM1, SSTR3, GABRG3, GABRG2, ATCAY, GRIN3A, SV2B, NPBWR1, CPEB3, CAMK1G	1.45E‐07
CC	GO:0005840~ribosome	10	RPS14, RPS15A, RPS16, RPL32, RPS29, RPL27A, RPS3, RPL35A, RPS3A, RPL9	1.91E‐05
CC	GO:0030424~axon	13	KCNC2, STXBP1, KCNAB2, HTR2A, OPRM1, GABRG2, DNM3, PAK1, ATCAY, SNCG, NEFM, HCN1, SNCA	7.62E‐05
CC	GO:0098982~GABA‐ergic synapse	7	GABRA1, SLC6A17, KCNC2, IQSEC3, ATP2B3, GABRG3, GABRG2	9.74E‐05
KEGG	hsa04080:Neuroactive ligand‐receptor interaction	19	GABRA1, CHRM1, GABRA6, GLP2R, HTR1A, HTR2C, GPR83, HTR2A, OPRM1, SSTR3, GABRG3, RXFP1, GABRG2, MAS1, GRM4, GRIN3A, HRH3, ADORA3, NPBWR1	8.47E‐08
KEGG	hsa04727:GABAergic synapse	9	PRKCG, GABRA1, GNG3, GNGT2, SLC12A5, GABRA6, GLS2, GABRG3, GABRG2	5.78E‐06
KEGG	hsa03010:Ribosome	11	RPS14, RPS15A, RPS16, RPL32, RPS29, RPL27A, RPS3, RPL35A, RPS3A, RPL9, RPL19	1.48E‐05
KEGG	hsa05032:Morphine addiction	8	PRKCG, GABRA1, GNG3, GNGT2, GABRA6, OPRM1, GABRG3, GABRG2	6.30E‐05
KEGG	hsa04726:Serotonergic synapse	8	PRKCG, TPH2, GNG3, GNGT2, HTR1A, HTR2C, HTR3B, HTR2A	2.75E‐04
KEGG	hsa04724:Glutamatergic synapse	7	PRKCG, GNG3, PPP3R1, GRM4, GNGT2, GRIN3A, GLS2	0.001701
KEGG	hsa04650:Natural killer cell mediated cytotoxicity	7	LAT2, PRKCG, PAK1, PPP3R1, FCER1G, SYK, ITGB2	0.00281
KEGG	hsa04020:Calcium signalling pathway	9	PRKCG, PPP3R1, CHRM1, GRIN3A, FGF9, ATP2B3, HTR2C, HTR2A, CAMK1G	0.006925
KEGG	hsa04024:cAMP signalling pathway	7	MAPK9, PAK1, CHRM1, GRIN3A, HTR1A, ATP2B3, CNGB1	0.038778
KEGG	hsa04010:MAPK signalling pathway	8	PRKCG, MAPK9, PAK1, PPP3R1, FGF9, RASGRF2, CACNA2D4, MAP3K9	0.05

**TABLE 3:** Functional enrichment analysis of genes that interact with MS4A6A in the PPI network.

Category	Term	Count	Genes	*p* Value
BP	GO:0007166~cell surface receptor signalling pathway	3	CD86, FCGR2A, MS4A7	0.0091958
BP	GO:0002224~toll‐like receptor signalling pathway	2	TLR8, CTSS	0.01244988
BP	GO:0032753~positive regulation of interleukin‐4 production	2	CD86, FCER1G	0.01244988
BP	GO:0032703~negative regulation of interleukin‐2 production	2	LAPTM5, VSIG4	0.01290833
BP	GO:0019886~antigen processing and presentation of exogenous peptide antigen via MHC class II	2	FCER1G, CTSS	0.01474024
BP	GO:0006955~immune response	3	CD86, CTSS, C1QC	0.02089994
BP	GO:0042130~negative regulation of T cell proliferation	2	CD86, VSIG4	0.022492168
BP	GO:0045087~innate immune response	3	FCER1G, TLR8, C1QC	0.032249129
BP	GO:1901224~positive regulation of NIK/NF‐kappaB signalling	2	CD86, LAPTM5	0.032443573
BP	GO:0016064~immunoglobulin mediated immune response	2	FCER1G, TLR8	0.039176889
MF	GO:0019864~IgG binding	2	FCGR2A, FCER1G	0.005792506
CC	GO:0016021~integral component of membrane	10	CD86, SLC7A7, FCGR2A, FCER1G, MS4A7, LAPTM5, TLR8, C1ORF162, VSIG4, MS4A4A	1.84E‐04
CC	GO:0005886~plasma membrane	8	CD86, SLC7A7, FCGR2A, FCER1G, MS4A7, LAPTM5, TLR8, MS4A4A	0.009202711
CC	GO:0009897~external side of plasma membrane	3	CD86, FCER1G, TLR8	0.02557098
CC	GO:0005887~integral component of plasma membrane	4	SLC7A7, FCGR2A, FCER1G, LAPTM5	0.036563874
KEGG	hsa05322:Systemic lupus erythematosus	3	CD86, FCGR2A, C1QC	0.00683036
KEGG	hsa05152:Tuberculosis	3	FCGR2A, FCER1G, CTSS	0.011572895

### 3.9 Validation of expression changes of seed nodes and their related lncRNAs

**FIGURE 8:**
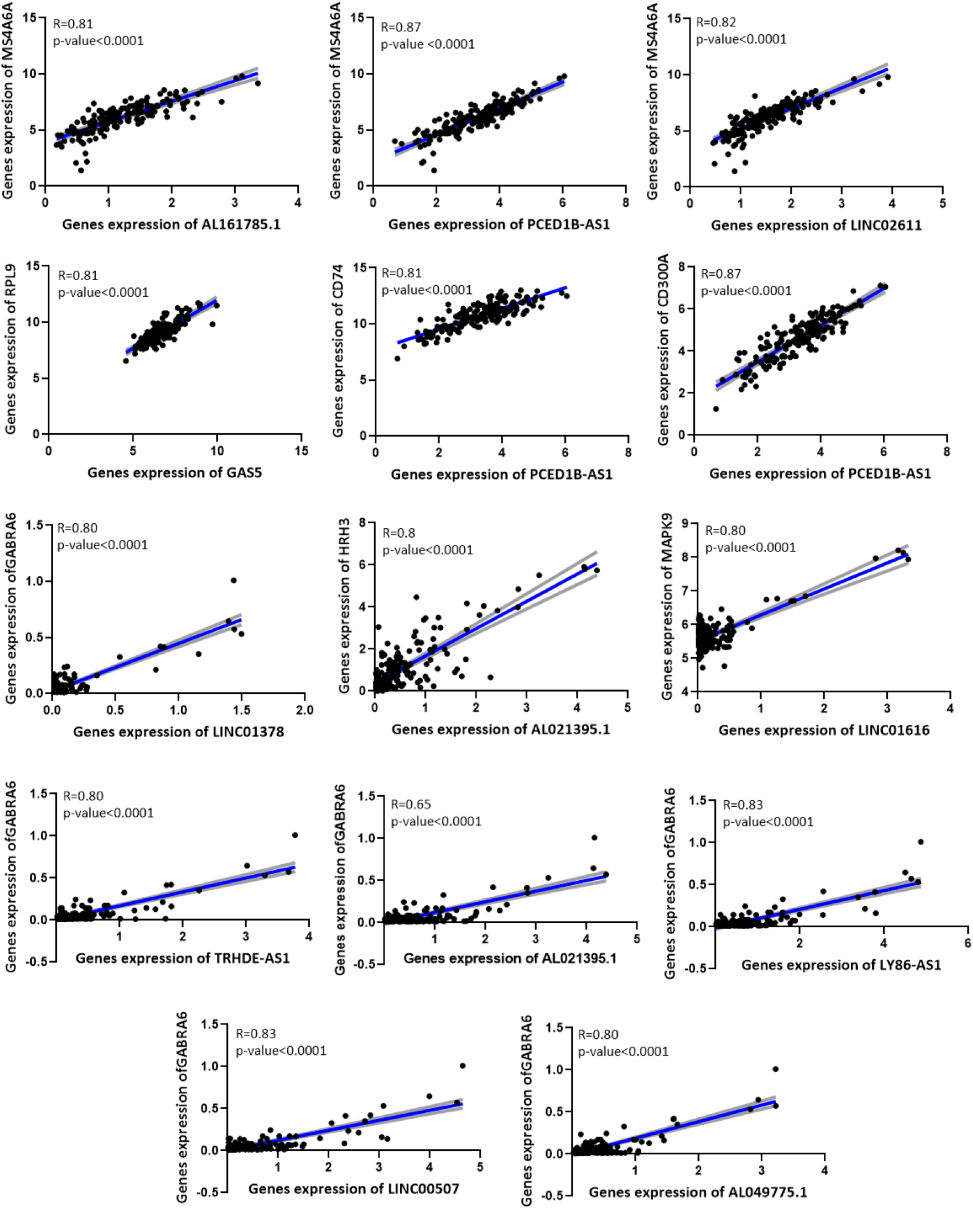
Positive correlation between seed nodes and their related lncRNAs. The connecting line is shown in blue, and the error bars are shown in grey.

**FIGURE 9:**
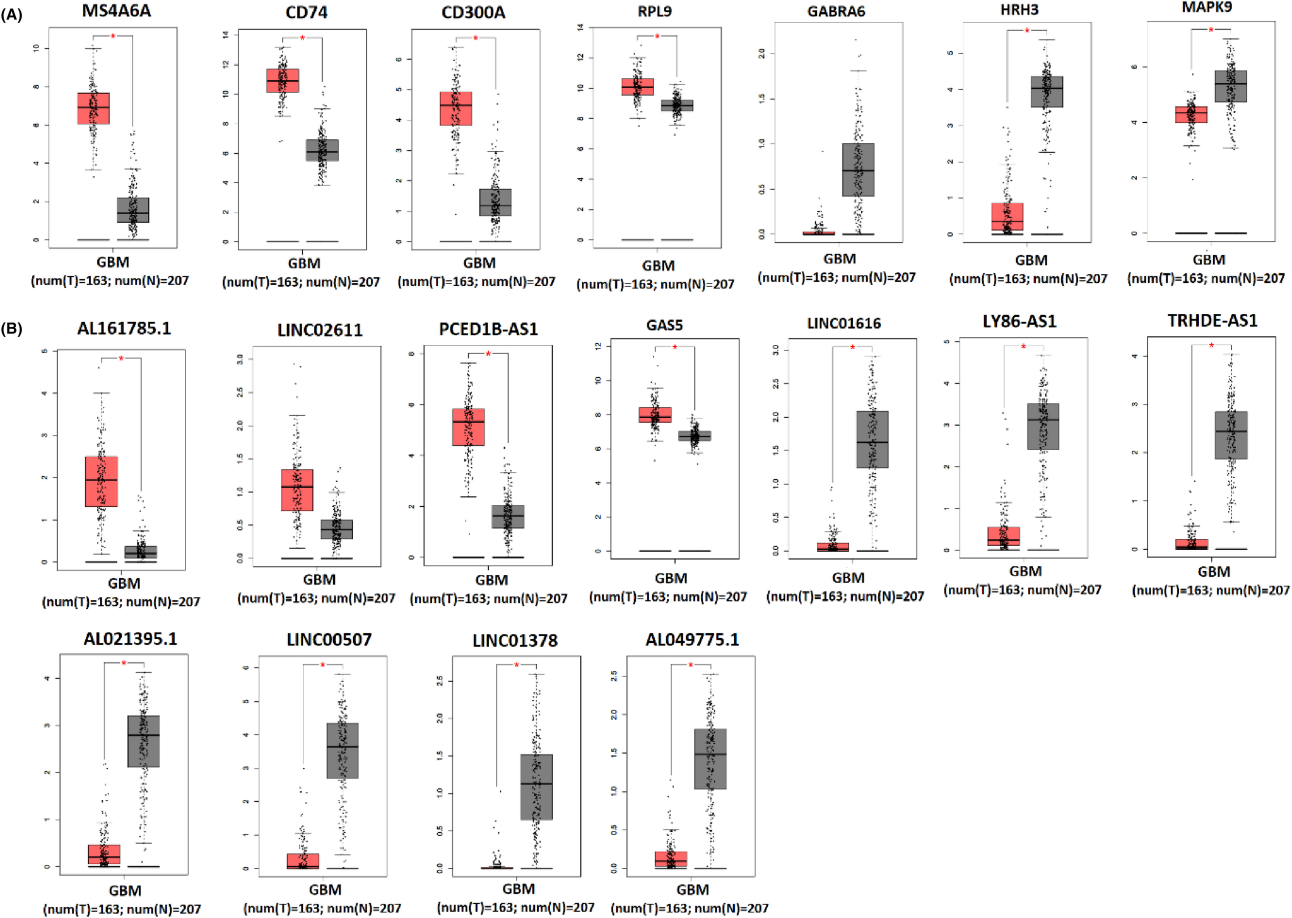
Validation of expression changes of seed nodes and their related lncRNAs in GBM tissue. (A) It represents up/downregulated mRNAs in biopsies of patients with GBM (T) (red box) compared to normal tissue (N) (grey box), including MS4A6A, CD74, CD300A, RPL9, GABRA6, HRH3, and MAPK9. (B) It shows up/downregulated lncRNAs in biopsies of patients with GBM (T) (red box) compared with normal tissue (N) (grey box), including AL161785.1, LINC02611, PCED1B‐AS1, GAS5, LINC01616, LY86‐AS1, TRHDE‐AS1, AL021395.1, LINC00507, LINC01378, AL049775.1. Data were obtained from the TCGA and GTEx datasets. *p < 0.01. Expression was log2 transformed (TPM + 1).

**FIGURE 10:**
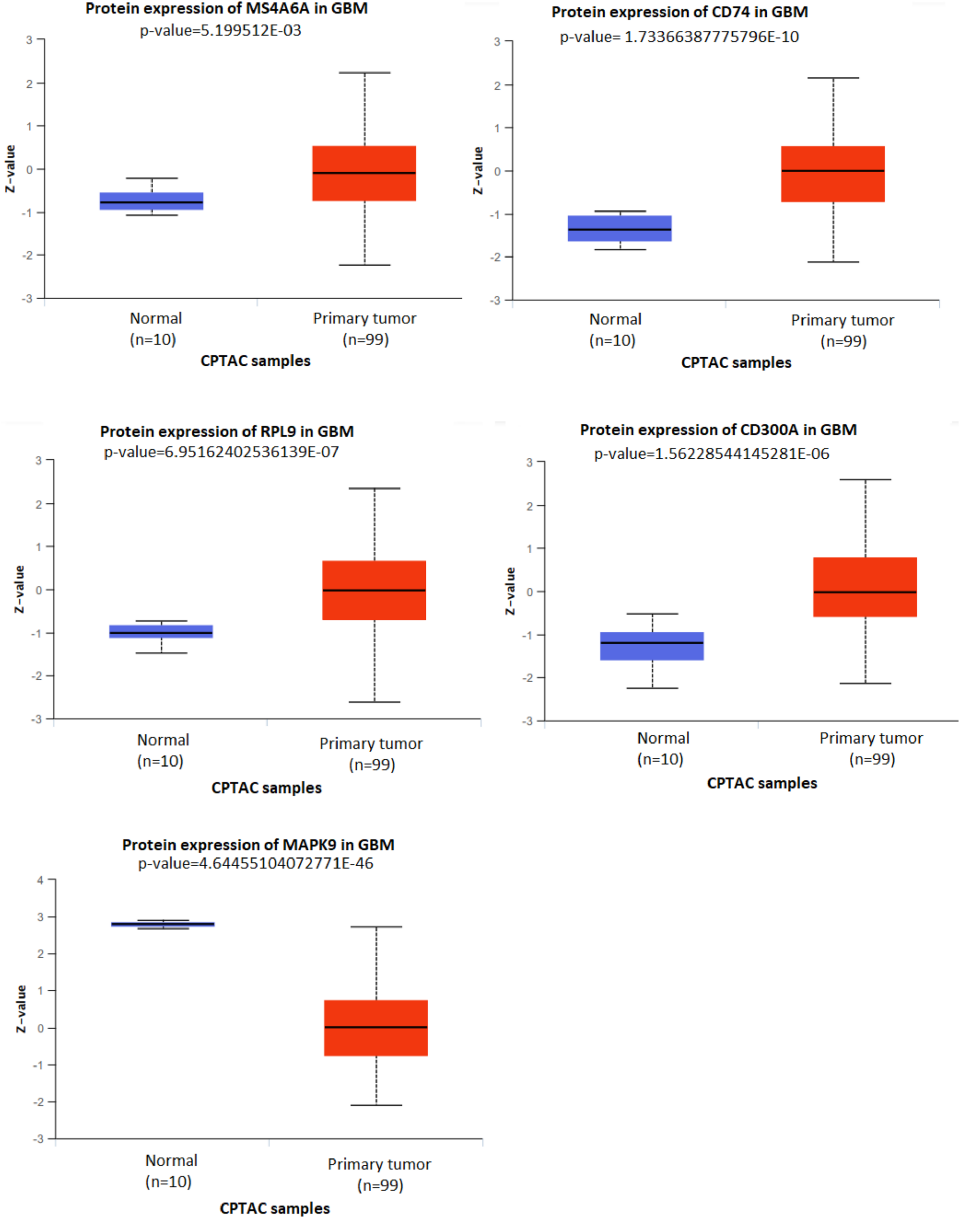
Validation of expression changes of hub genes at the protein level in GBM tissue using the CPTAC database.

Figure 8 shows that our findings showed a positive correlation between seed nodes and their related lncRNAs, validated with the GEPIA database (File S9). We found that four seed genes (MS4A6A, CD74, CD300A and RPL9) and their related lncRNAs were upregulated in GBM, while the expression of the other three seed nodes (MAPK9, HRH3 and GABRA6) and their regulatory lncRNAs decreased in GBM (Files S2 and S3). The expression level of these molecules in GBM tissues compared with normal samples was validated at the RNA level using the GEPIA database, shown in Figure 9. In addition, with the help of the CPTAC database, we showed that the expression of four seed genes (MS4A6A, CD74, CD300A and RPL9) and MAPK9 were also increased and decreased at the protein level in GBM (Figure 10).

### 3.10 Identification of possible critical protein‐coding and non‐coding RNAs

Finally, to find possible critical protein‐coding and non‐coding RNAs involved in GBM, we intersected between the hub genes of the ceRNA network and the lncRNA‐miRNA‐seed network constituent nodes. As shown in Table 4, hsa‐miR‐432‐5p, hsa‐miR‐485‐5p, hsa‐miR‐433‐3p, hsa‐miR‐4701‐3p, hsa‐miR‐27a‐3p, hsa‐miR‐139‐5p, hsa‐miR‐15b‐5p, LINC00507 and GAS5 were shared between two gene groups, which may play a key role in GBM pathogenesis. Among these common genes, two microRNAs (hsa‐miR‐433‐3p and hsa‐miR‐139‐5p) regulate the MS4A6A expression, which before mentioned increasing its expression is related to a poor prognosis (Figure 6). Our study showed that the expression of these two microRNAs had a 16‐fold decrease in GBM compared with the normal group (File S4); on the other hand, the expression of their target gene (MS4A6A) had a 16‐fold increase in GBM (Table 1 and File S2). In addition, our study showed that hsa‐miR‐139‐5p is regulated by two lncRNAs (AL161785.1 and LINC02611), and hsa‐miR‐433‐3p is regulated by PCED1B‐AS1 lncRNA, which these three lncRNAs are positively correlated with MS4A6A (Figure 8). These results suggest that these regulatory axes probably play an essential role in GBM pathogenesis and progression.

**TABLE 4:** Possible critical protein‐coding and non‐coding RNAs involved in GBM.

Hubgenes in ceRNA network (A)	hsa‐miR‐210‐3p, hsa‐miR‐4701‐3p, hsa‐miR‐27a‐3p, hsa‐miR‐15b‐5p, hsa‐miR‐485‐5p, hsa‐miR‐195‐5p, hsa‐miR‐23a‐3p, hsa‐miR‐139‐5p, hsa‐miR‐497‐5p, hsa‐miR‐432‐5p, GAS5, hsa‐miR‐433‐3p, hsa‐let‐7 g‐5p, PART1, APBA1, hsa‐miR‐98‐5p, SNHG29, LINC00507, LINC00641, PABPC1L2A, HTR3B, SEC31B, GPR26, PTER, RPL32
Nodes forming the lncRNA‐miRNA‐seed network (B)	AL161785.1, GAS5, PCED1B‐AS1, LINC02611, AL021395.1, LY86‐AS1, LINC00507, LINC01378, AL049775.1, LINC01616, TRHDE‐AS1, hsa‐miR‐139‐5p, hsa‐miR‐432‐5p, hsa‐miR‐433‐3p, hsa‐miR‐485‐5p, hsa‐miR‐27a‐3p, hsa‐miR‐4701‐3p, hsa‐miR‐15b‐5p, MS4A6A, RPL9, CD74, CD300A, GABRA6, HRH3, MAPK9
A & B intersection (Critical genes in GBM)	hsa‐miR‐432‐5p, hsa‐miR‐485‐5p, hsa‐miR‐433‐3p, hsa‐miR‐4701‐3p, hsa‐miR‐27a‐3p, hsa‐miR‐139‐5p, hsa‐miR‐15b‐5p, LINC00507, GAS5
Seed node associated with overall survival	MS4A6A
MS4A6A‐related regulatory axes	AL161785.1/miR‐139‐5p/MS4A6A, LINC02611/miR‐139‐5p/MS4A6A, PCED1B‐AS1/miR‐433‐3p/MS4A6A

### 3.11 Correlation between MS4A6A expression and immune infiltration

As shown in Table 5, the MS4A6A expression level based on the GSCA database in GBM was significantly correlated positively with infiltration of Macrophage, Cytotoxic T cells, DC, Th2, NK, Th1, Effector memory T cells, Tfh, MAIT, iTreg, Exhausted T cells, CD8 T cells, nTreg, Tr1, and monocyte and negatively was associated with infiltration of CD4 T cells, Central memory T cells, CD4 naïve, Gamma delta T cells, Bcell, CD8 naïve, and Neutrophil. Our study showed a strong positive correlation between macrophage infiltration and MS4A6A expression in GBM (*R* = 0.85, Adjusted *p*‐value = 1.79714E‐47). Furthermore, MS4A6A is highly negatively correlated with neutrophil infiltration (*R* = −0.66, Adjusted *p*‐value = 8.424547599E‐21).

**TABLE 5:** Correlation between MS4A6A expression and immune infiltration in GBM.

Cancer type	Gene symbol	Cell type	Correlation	Adjusted *p*‐value
GBM	MS4A6A	Macrophage	0.851801651	1.79714E‐47
GBM	MS4A6A	InfiltrationScore	0.795467852	8.43588E‐37
GBM	MS4A6A	Cytotoxic T cells	0.639508481	1.37998E‐18
GBM	MS4A6A	DC	0.637502195	1.94521E‐18
GBM	MS4A6A	Th2	0.614471472	1.03042E‐16
GBM	MS4A6A	NK	0.518689671	6.55957E‐11
GBM	MS4A6A	Th1	0.510135043	1.38419E‐10
GBM	MS4A6A	Effector_memory	0.500276956	5.31189E‐10
GBM	MS4A6A	Tfh	0.440529107	1.14415E‐07
GBM	MS4A6A	MAIT	0.439351657	2.18845E‐07
GBM	MS4A6A	iTreg	0.422302973	6.81973E‐07
GBM	MS4A6A	Exhausted T cells	0.405057889	2.28196E‐06
GBM	MS4A6A	CD8_T	0.341912502	0.000182327
GBM	MS4A6A	nTreg	0.305395818	0.000888627
GBM	MS4A6A	Tr1	0.220916326	0.048472034
GBM	MS4A6A	Monocyte	0.205508664	0.026407288
GBM	MS4A6A	NKT	0.187844522	0.110836675
GBM	MS4A6A	Th17	0.020766137	0.877631962
GBM	MS4A6A	CD4_T	−0.052829334	0.602480294
GBM	MS4A6A	Central_memory	−0.216388205	0.03845588
GBM	MS4A6A	CD4_naive	−0.239732006	0.008443198
GBM	MS4A6A	Gamma_delta T cells	−0.366720811	0.000043399
GBM	MS4A6A	Bcell	−0.476683359	1.98869E‐09
GBM	MS4A6A	CD8_naive	−0.597260859	5.55781E‐16
GBM	MS4A6A	Neutrophil	−0.664402839	8.42455E‐21

## 4 DISCUSSION

Although glioblastoma research has developed rapidly over the past few decades, the specific molecular mechanisms are still unclear. Recently, more data have suggested that lncRNA dysregulation participates in tumour initiation and malignant progression.^29^ Thus, lncRNAs detected in tumour tissues could serve as candidate diagnostic biomarkers and therapeutic targets for GBM. However, the roles and mechanisms of aberrantly expressed lncRNAs in the pathogenesis of GBM are not fully deciphered yet, and more lncRNAs need to be uncovered. lncRNAs are a class of competing endogenous ncRNAs that can inhibit the binding of miRNAs to target mRNA and regulate the expression level of target genes by exerting a miRNA sequestering effect.^30^ In this study, we used the RNA and miRNA expression profiles from the TCGA and GEO datasets to construct deregulated lncRNAs‐associated networks of GBM based on the ceRNA theory. Our analysis revealed that three regulatory axes AL161785.1/miR‐139‐5p/MS4A6A, LINC02611/miR‐139‐5p/MS4A6A and PCED1B‐AS1/miR‐433‐3p/MS4A6A might play essential roles in disease pathogenesis. MS4A6A has been recognized as being associated with ageing‐related neurodegenerative disease^31^, ^32^, ^33^, ^34^ and cancer.^35^, ^36^ MS4A6A was reported to be highly expressed in putative tumour‐associated macrophage (TAM) populations.^37^, ^38^ TAMs are heterogeneous populations that include brain‐resident microglia, border‐associated macrophages (BAMs), and bone marrow‐derived macrophages (BMDMs). TAMs correlate negatively with infiltration of T cells, neutrophils and plasmacytoid dendritic cells (pDCs), leading to the immunosuppressive nature of the glioma immune microenvironment (GIME).^39^, ^40^ Therefore, TAMs can promote tumorigenesis by suppressing immune surveillance and inducing angiogenesis.^37^, ^39^ Furthermore, studies have shown that the pathological grade in glioma increases with the increase of TAM accumulations in tumour tissue, indicating the critical role of TAMs in tumour development.^41^, ^42^ A recent study found that MS4A6A overexpression in GBM tissue may promote macrophage infiltration in the GIME, leading to immunosuppression.^38^ The present study also found a strong correlation between MS4A6A expression and macrophage infiltration. Furthermore, studies showed a positive correlation between the MS4A6A expression and the WHO grades of glioma, suggesting its possible role in GBM development.^38^ In addition, it has been identified that high expression of MS4A6A in glioma tissue is associated with a worse prognosis in patients,^38^ which is consistent with the result of this study. Our study demonstrated that MS4A6A expression has a positive correlation with MS4A4A, VSIG4, C1QC, FCER1G and CD86 expression, which may explain why MS4A6A participates in the induction of macrophage infiltration and immune suppression. studies showed that the MS4A4A,^43^, ^44^ VSIG4,^43^, ^45^ C1QC^46^ FCER1G^47^ and CD86^48^ genes were highly expressed in TAMs and related to macrophage infiltration in cancer. Furthermore, functional annotation of MS4A6A‐related genes confirmed the tight correlation of MS4A6A with the immune response. However, more studies are needed to fully understand the relationship between MS4A6A, macrophage infiltration and GBM. Our study revealed the competing regulatory associations between the three lncRNAs (AL161785.1, PCED1B‐AS1, and LINC02611) and the two miRNAs (hsa‐miR‐139‐5p, hsa‐miR‐433‐3p) with the MS4A6A in GBM. The results of the present study showed that the expression of miR‐139‐5p and miR‐433‐3p is decreased in GBM, which was in line with previous studies.^49^, ^50^, ^51^ Evidence showed that miR‐139‐5p acts as a tumour suppressor by targeting ELTD1 and regulating the cell cycle in GBM,^50^ targeting GABRA1,^52^ EIF4G2,^53^ ZEB1 and ZEB2^54^ in GBM. Furthermore, it has been reported that miR‐433 can inhibit GBM progression by suppressing the PI3K/Akt signalling pathway through direct targeting of ERBB4.^55^ The ceRNA network shows that AL161785.1 and LINC02611 regulates MS4A6A expression by sponging miR‐139‐5p, and PCED1B‐AS1 regulates MS4A6A expression by sponging miR‐433‐3p. We observed that the expression of these three lncRNAs and MS4A6A in GBM were increased compared with normal tissue. In addition, we also observed a strong positive correlation between these three lncRNAs and MS4A6A expression in GBM tissues. These results suggest that these three regulatory axes probably play an important role in GBM pathogenesis and progression. There is evidence that PCED1B‐AS1 was significantly overexpressed in GBM and promotes tumorigenesis by upregulating HIF‐1α in GBM.^56^ Studies have shown that knockdown of PCED1B‐AS1 inhibits the Warburg effect and cell proliferation, while overexpression of PCED1B‐AS1 results in opposite effects.^56^ So far, no study has reported the contribution of AL161785.1 and LINC02611 in GBM development, but it has been identified that AL161785.1 is related to shorter survival time of patients with Stomach Adenocarcinoma^57^ and LINC02611 can be used as a poor prognostic biomarkers in renal clear cell carcinoma.^58^ Taken together, our findings demonstrate that AL161785.1 and LINC02611 are novel oncogenic lncRNAs in GBM and could regulate MS4A6A expression, which provides a promising prognostic biomarker, by sponging miR‐139‐5p. While the present study offers valuable insights into the lncRNA‐associated ceRNA network in GBM, we must acknowledge certain limitations inherent in our methodology. Primarily, our investigation was conducted in silico, relying on computational predictions and analyses. Therefore, further experimental validation of these newly identified regulatory axes seems promising to confirm their biological relevance and functional significance. For future studies, employing practical techniques such as dual‐luciferase reporter assay seems beneficial to validate the interactions between lncRNA‐miRNA and miRNA‐mRNA. Moreover, the use of miR‐139‐5p and miR‐433‐3p mimics or inhibitors could modulate the expression of the identified lncRNAs (AL161785.1, LINC02611 and PCED1B‐AS1) and could help elucidate their potential impact on MS4A6A expression. Hence, addressing these limitations through rigorous experimental validation in future studies would be valuable and would strengthen the reliability and significance of our findings, facilitating their translation into potential clinical usage. Finally, as previously stated, the WHO 2021 CNS classification (CNS WHO5) categorizes adult diffuse gliomas grade IV into IDH1 wildtype glioblastoma and grade IV IDH1‐mutant astrocytoma. Nevertheless, due to the fact that databases like TCGA or GEPIA, which were utilized for our research, as well as current academic literature, identify these tumours as glioblastoma, we have chosen to refer to them as such in this study.

## 5 CONCLUSIONS

Comprehensive analysis of lncRNA‐associated ceRNA network identified that three novel potential prognostic regulatory axes (AL161785.1/miR‐139‐5p/MS4A6A, LINC02611/miR‐139‐5p/MS4A6A, and PCED1B‐AS1/miR‐433‐3p/MS4A6A) probably play an essential role in GBM pathogenesis and progression. Therefore, targeting them may be a promising approach for treating GBM patients.

## AUTHOR CONTRIBUTIONS

**Maryam Bazrgar:** Conceptualization (lead); data curation (lead); formal analysis (lead); investigation (lead); methodology (lead); project administration (supporting); software (equal); validation (lead); writing – original draft (lead). **Seyed Amir Mirmotalebisohi:** Formal analysis (supporting); methodology (supporting); software (supporting); validation (supporting); writing – review and editing (equal). **Mohsen Ahmadi:** Methodology (supporting); supervision (supporting); writing – review and editing (equal). **Parisa Azimi:** Supervision (supporting); writing – review and editing (equal). **Leila Dargahi:** Supervision (supporting); writing – review and editing (equal). **Hakimeh Zali:** Conceptualization (lead); methodology (lead); project administration (supporting); supervision (supporting); validation (lead); visualization (lead); writing – review and editing (equal). **Abolhassan Ahmadiani:** Conceptualization (lead); funding acquisition (lead); project administration (supporting); validation (supporting); writing – review and editing (equal).

## FUNDING INFORMATION

This study was supported by a grant from Shahid Beheshti University of Medical Sciences (No. 43005002‐7‐4).

## CONFLICT OF INTEREST STATEMENT

The authors declare that they have no conflict of interest.

## Supporting information


File S1.

File S2.

File S3.

File S4.

File S5.

File S6.

File S7.

File S8.

File S9.

File S10.

## Data Availability

Data openly available in a public repository that issues datasets with DOIs.
