# Supplementary material for: Comprehensive analysis of lncRNA‐associated ceRNA network reveals novel potential prognostic regulatory axes in glioblastoma multiforme
Source: J Cell Mol Med. 2024 Jun 12;28(11):e18392. doi: 10.1111/jcmm.18392 (PMC11167707; doi:10.1111/jcmm.18392)
Supplement: Supplementary file 9 — File S9. [file JCMM-28-e18392-s003.docx]

**Positive correlation between 12 genes expression (CD86, C1QC, C1orf162, CTSS, FCER1G, FCGR2A, LAPTM5, MS4A7, SLC7A7, VSIG4, TLR8, and MS4A4A) and MS4A6A expression using the GEPIA database**


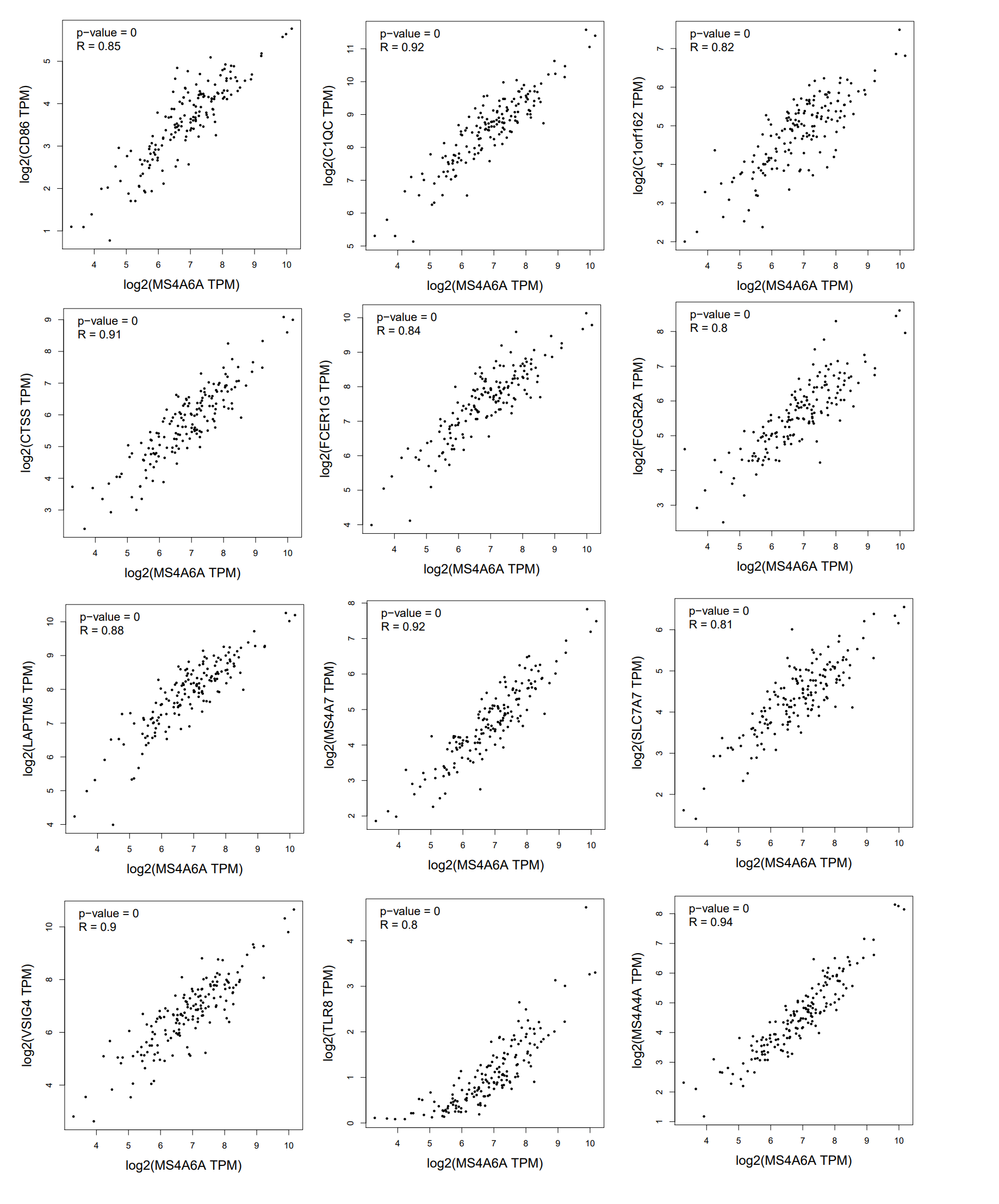


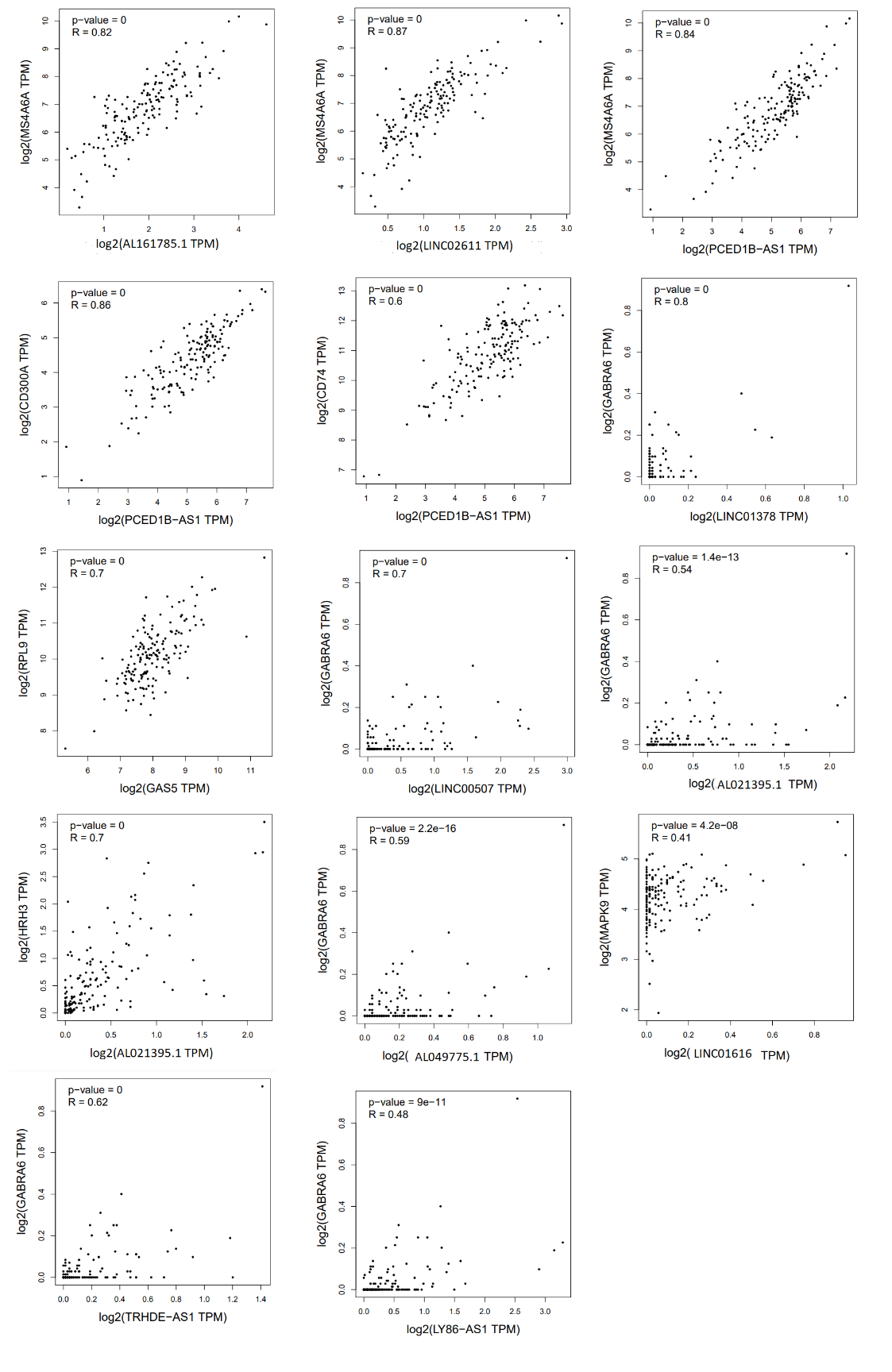
**Validation of positive correlation between seed nodes and their related lncRNAs using the GEPIA database**
